# Supplementary material for: Examining multiple cellular pathways at once using multiplex hextuple luciferase assaying
Source: Nat Commun. 2019 Dec 13;10:5710. doi: 10.1038/s41467-019-13651-y (PMC6911020; doi:10.1038/s41467-019-13651-y)
Supplement: Supplementary file 3 — Description of Additional Supplementary Files [file 41467_2019_13651_MOESM3_ESM.pdf]

Description of Additional Supplementary Files

File name: Supplementary Data 1

Description: Excel file containing four worksheets to facilitate the analysis of luminescence emission data obtained by a microplate reader for multiplex hextuple luciferase assaying. **Worksheet 1**: protected read-only Excel worksheet containing formulas for the calculation of transmission coefficients (explained further in **Supplementary Figure 23**). **Worksheet 2**: protected read-only Excel worksheet containing formulas for the calculation of simultaneous equations (explained further in **Supplementary Figure 24**). **Worksheet 3**: protected read-only Excel worksheet containing formulas for the unformatted measurements from a small group of samples up to 12 (explained further in **Supplementary Figure 25**). **Worksheet 4**: protected read-only Excel worksheet containing formulas for the unformatted measurements from a large group of samples up to an entire 96-well plate (explained further in **Supplementary Figure 26**). Analyzed data was then migrated into Prism 7 software (GraphPad) for statistical analysis and graphing. The resulting graphs were then edited for publication using Adobe Illustrator CC (Adobe Creative Cloud) (see **Methods**).
