# Supplementary material for: Examining multiple cellular pathways at once using multiplex hextuple luciferase assaying
Source: Nat Commun. 2019 Dec 13;10:5710. doi: 10.1038/s41467-019-13651-y (PMC6911020; doi:10.1038/s41467-019-13651-y)
Supplement: Supplementary file 5 — Reporting Summary [file 41467_2019_13651_MOESM5_ESM.pdf]

nature research

Corresponding author(s): Koen Venken

Last updated by author(s): Nov 1, 2019

Reporting Summary

Nature Research wishes to improve the reproducibility of the work that we publish. This form provides structure for consistency and transparency in reporting. For further information on Nature Research policies, see Authors & Referees and the Editorial Policy Checklist.

Statistics

For all statistical analyses, confirm that the following items are present in the figure legend, table legend, main text, or Methods section.

n/a	Confirmed	
☐	☒	The exact sample size (n) for each experimental group/condition, given as a discrete number and unit of measurement
☐	☒	A statement on whether measurements were taken from distinct samples or whether the same sample was measured repeatedly
☐	☒	The statistical test(s) used AND whether they are one- or two-sided<br>*Only common tests should be described solely by name; describe more complex techniques in the Methods section.*
☒	☐	A description of all covariates tested
☒	☐	A description of any assumptions or corrections, such as tests of normality and adjustment for multiple comparisons
☐	☒	A full description of the statistical parameters including central tendency (e.g. means) or other basic estimates (e.g. regression coefficient) AND variation (e.g. standard deviation) or associated estimates of uncertainty (e.g. confidence intervals)
☐	☒	For null hypothesis testing, the test statistic (e.g. F, t, r) with confidence intervals, effect sizes, degrees of freedom and P value noted<br>*Give P values as exact values whenever suitable.*
☒	☐	For Bayesian analysis, information on the choice of priors and Markov chain Monte Carlo settings
☒	☐	For hierarchical and complex designs, identification of the appropriate level for tests and full reporting of outcomes
☒	☐	Estimates of effect sizes (e.g. Cohen's d, Pearson's r), indicating how they were calculated

Our web collection on statistics for biologists contains articles on many of the points above.

Software and code

Policy information about availability of computer code

Data collection	For luminescence, we obtained data with the MARS Data Analysis Software built in the CLARIOStar Luminometer (BMG). For qPCR, we obtained data with the LightCycler® 480 built in Software (Roche).
Data analysis	We used Excel and Graphpad Prism for data analysis.

For manuscripts utilizing custom algorithms or software that are central to the research but not yet described in published literature, software must be made available to editors/reviewers. We strongly encourage code deposition in a community repository (e.g. GitHub). See the Nature Research guidelines for submitting code & software for further information.

Data

Policy information about availability of data

All manuscripts must include a data availability statement. This statement should provide the following information, where applicable:

- Accession codes, unique identifiers, or web links for publicly available datasets
- A list of figures that have associated raw data
- A description of any restrictions on data availability

Addgene plasmid/culture coordinates are included in the paper.

Field-specific reporting

Please select the one below that is the best fit for your research. If you are not sure, read the appropriate sections before making your selection.

☒ Life sciences ☐ Behavioural & social sciences ☐ Ecological, evolutionary & environmental sciences

Life sciences study design

All studies must disclose on these points even when the disclosure is negative.

Sample size	All multiplex luciferase and qPCR experiments consisted of 6 biological replicates. Experiments to determine the compatibility of the luciferases consisted on 6 technical replicates.
Data exclusions	Outlier data points were excluded, exceding +/- 2 times the standard deviation of the assay and luminiscence data with values out of the discussed dynamic range were also excluded, to a maximum of 2 data points per experiment, resulting in n = 4 for each experiment.
Replication	We replicated successfully all experiments in the manuscript. siRNA, drugs and ligand treatments to the cells were simultaneously analyzed by the multiplex luciferase assay and by qPCR and data showed the same response. Both analysis are included in the manuscript.
Randomization	Randomization was not relevant to this work.
Blinding	Blinding was not relevant to this work.

Reporting for specific materials, systems and methods

We require information from authors about some types of materials, experimental systems and methods used in many studies. Here, indicate whether each material, system or method listed is relevant to your study. If you are not sure if a list item applies to your research, read the appropriate section before selecting a response.

Materials & experimental systems

n/a	Involved in the study
☒	☐ Antibodies
☐	☒ Eukaryotic cell lines
☒	☐ Palaeontology
☒	☐ Animals and other organisms
☒	☐ Human research participants
☒	☐ Clinical data

Methods

n/a	Involved in the study
☒	☐ ChIP-seq
☒	☐ Flow cytometry
☒	☐ MRI-based neuroimaging

Eukaryotic cell lines

Policy information about cell lines

Cell line source(s)	All cell lines used in this study were obtained from the Tissue and Cell Culture Core at Baylor College of Medicine and the Characterized Cell Line Core Facility at MD Anderson.
Authentication	Short tandem repeat DNA fingerprinting was performed at the Characterized Cell Line Core Facility (MD Anderson).
Mycoplasma contamination	Cell lines were tested negative for mycoplasma contamination.
Commonly misidentified lines (See ICLAC register)	There were no misidentified lines.
